# Pangenomic analysis of *Coxiella burnetii* unveils new traits in genome architecture

**DOI:** 10.3389/fmicb.2022.1022356

**Published:** 2022-11-21

**Authors:** Rita Abou Abdallah, Matthieu Million, Jeremy Delerce, Hussein Anani, Awa Diop, Aurelia Caputo, Rita Zgheib, Elodie Rousset, Karim Sidi Boumedine, Didier Raoult, Pierre-Edouard Fournier

**Affiliations:** ^1^Aix Marseille Université, Institut de Recherche pour le Développement (IRD), Service de Santé des Armées, AP-HM, UMR Vecteurs Infections Tropicales et Méditerranéennes (VITROME), Institut Hospitalo-Universitaire Méditerranée Infection, Marseille, France; ^2^Institut Hospitalo-Universitaire Méditerranée Infection, Marseille, France; ^3^Aix-Marseille Université, Institut de Recherche pour le Développement (IRD), UMR Microbes Evolution Phylogeny and Infections (MEPHI), Institut Hospitalo-Universitaire Méditerranée Infection, Marseille, France; ^4^French Agency for Food, Environmental and Occupational Health Safety (ANSES), Sophia Antipolis Laboratory, Animal Q Fever Unit, Sophia Antipolis, France

**Keywords:** *Coxiella burnetii*, Q fever, pangenome, genotyping, pathogenesis

## Abstract

*Coxiella burnetii* is the etiological agent of Q fever, a worldwide zoonosis able to cause large outbreaks. The disease is polymorphic. Symptomatic primary infection is named acute Q fever and is associated with hepatitis, pneumonia, fever, and auto-immune complications while persistent focalized infections, mainly endocarditis, and vascular infections, occur in a minority of patients but are potentially lethal. In order to evaluate the genomic features, genetic diversity, evolution, as well as genetic determinants of antibiotic resistance, pathogenicity, and ability to cause outbreaks of Q fever, we performed a pangenomic analysis and genomic comparison of 75 *C. burnetii* strains including 63 newly sequenced genomes. Our analysis demonstrated that *C. burnetii* has an open pangenome, unique genes being found in many strains. In addition, pathogenicity islands were detected in all genomes. In consequence *C. burnetii* has a high genomic plasticity, higher than that of other intracellular bacteria. The core- and pan-genomes are made of 1,211 and 4,501 genes, respectively (ratio 0.27). The core gene-based phylogenetic analysis matched that obtained from multi-spacer typing and the distribution of plasmid types. Genomic characteristics were associated to clinical and epidemiological features. Some genotypes were associated to specific clinical forms and countries. MST1 genotype strains were associated to acute Q fever. A significant association was also found between clinical forms and plasmids. Strains harboring the QpRS plasmid were never found in acute Q fever and were only associated to persistent focalized infections. The QpDV and QpH1 plasmids were associated to acute Q fever. In addition, the Guyanese strain CB175, the most virulent strain to date, exhibited a unique MST genotype, a distinct COG profile and an important variation in gene number that may explain its unique pathogenesis. Therefore, strain-specific factors play an important role in determining the epidemiological and clinical manifestations of Q fever alongside with host-specific factors (valvular and vascular defects notably).

## Introduction

*Coxiella burnetii*, an obligate Gram-negative and strictly intracellular bacterium responsible for the zoonotic disease Q fever (Angelakis and Raoult, 2010), was first discovered in 1935 during an outbreak of febrile illness in abattoir workers in Brisbane, Australia (Derrick, 1983). Two years later, *C. burnetii* was first isolated after injecting patient blood and urine samples to guinea pigs (Angelakis and Raoult, 2010). It was initially classified as a member of the genus *Rickettsia* (Angelakis and Raoult, 2010), prior to being reclassified into the new genus *Coxiella* in 1948, after studying its cultural and biochemical characteristics. Currently, Q fever is endemic worldwide, except in New Zealand (Hilbink et al., 1993). The disease occurs mostly as sporadic infections, although outbreaks have been reported in France (Frankel et al., 2011), Great Britain (Smith et al., 1993), Italy (Manfredi Selvaggi et al., 1996), Germany (Lyytikäinen et al., 1998), and the Netherlands (de Bruin et al., 2012). The largest outbreak occurred from 2007 to 2012 in the latter country, with more than 4,000 diagnosed cases (de Bruin et al., 2012).

Even though its animal reservoir is large, domestic ruminants such as goats, sheep and less frequently cattle, represent the most common source of Q fever. *Coxiella burnetii* is mostly transmitted to humans by aerosols from parturition fluids of infected animals or from microorganisms spread by the wind. The bacterium is very resistant and may survive several weeks in the environment. Although *C. burnetii* was detected in ticks, this mode of transmission remains rare (Angelakis and Raoult, 2010).

In animals, Q fever is almost asymptomatic with the exception of cases of abortion, low birth weight and infertility (Angelakis and Raoult, 2010). In contrast to animal infection, human Q fever is characterized by a polymorphic clinical presentation (Raoult et al., 2005; Eldin et al., 2017; Melenotte et al., 2020). In acute, or primary Q fever, symptoms can range from an asymptomatic sero-conversion to a flu-like self-limited illness, to more severe symptoms including interstitial pneumonia (Caron et al., 1998), granulomatous hepatitis and rarely cardiac involvement (Fournier et al., 2001), skin rash and neurological signs (Angelakis et al., 2012). Patients may subsequently develop persistent focalized infections defined by both the microbe multiplication and the presence of organic lesions (Raoult, 2017). Endocarditis is the most frequent persistent focalized infection with *C. burnetii* (Angelakis and Raoult, 2010) involving in priority the aortic and mitral valves as well as valvular prostheses. Vascular infections are the second most frequent persistent focalized infection (O’Donnell et al., 2007; Angelakis and Raoult, 2010; Million and Raoult, 2017) and have a more severe prognosis than endocarditis. Other, less frequent manifestations of persistent focalized Q fever include osteoarticular infections (Angelakis et al., 2014), pseudo-tumors of the lung and persistent lymphadenitis that may result in lymphoma (Melenotte et al., 2016). It was proposed that the clinical presentation, host type and severity of Q fever may vary depending on the involved *C. burnetii* strain (Glazunova et al., 2005; Roest et al., 2011; de Bruin et al., 2012; Angelakis et al., 2013).

The availability of whole *C. burnetii* genome sequences was a major step forward for studying the pathophysiology of Q fever (Glazunova et al., 2005; D’Amato et al., 2014, 2016; Kuley et al., 2017). The first *C. burnetii* genome was sequenced from Nine Mile Phase I RSA493 (NM-I) strain in 2003. The 1,995,275-bp-long NM-I genome with a 37,393 QpH1 plasmid had mobile elements and had different metabolic capabilities, transport profile and had limited extent of genome reduction compared to obligate intracellular pathogens like *Rickettsia, Chlamydia*, and *Mycobacterium leprae* suggesting comparatively recent intracellular adaptation (Seshadri et al., 2003). Over recent years, thanks to the development of high throughput sequencing methods, 14 additional *C. burnetii* strain genomes were sequenced and allowed comparative genomic analyses. Prior to our study, the size and G + C content of *C. burnetii* genomes ranged from 1,956,650 to 2,093,477 bp and from 42.5 to 42.9%, respectively (Kuley et al., 2017). Four plasmid types were also identified (QpH1, QpRs, QpDv, and QpDg), with strains exhibiting any one or none of them (Beare et al., 2009; Sidi-Boumedine et al., 2014). Samuel et al. (1985) studied the correlation between plasmid type and disease presentation (acute or persistent focalized) and suggested that plasmid types may be associated to distinct clinical forms. Plasmid type has also been associated with pregnancy outcome (Angelakis et al., 2013). Beare et al. (2009) proposed that *C. burnetii* strains are at various stages of pathoadaptation. They characterized the genomic features associated to the obligate intracellular lifestyle of this pathogen such as genome reduction, presence of pseudogenes, and mobile genetic elements (Beare et al., 2009). The role of up-regulated genes in the survival and replication of the bacterium in hosts was also investigated (Kuley et al., 2015). These authors suggested that the Dugway strain was the most primitive due to its large genome and few pseudogenes and insertion sequences, in addition to the fact that it has the shortest distance to the root of the phylogenetic tree (Beare et al., 2009). By studying the virulent strain Guyane CB175, D’Amato et al. (2015) showed that its genome exhibited a 6,105 bp deletion and a large number of gene mutations compared to strain NM-I. Other genomic comparisons aimed at deciphering the high virulence of some strains such as Guyane CB175 and those responsible for the German outbreaks (D’Amato et al., 2014, 2015; Kuley et al., 2017). As whole-genome sequencing and comparative genome analysis of a large number of related strains has recently emerged as a cost-effective and convenient method allowing a better identification of the diversity and composition of the global gene repertoire of a bacterium (Tettelin et al., 2008), we performed a pangenomic analysis of *C. burnetii* using the genomes from 75 *C. burnetii* strains including 63 novel genomes, in order to evaluate the genetic diversity, evolution, antibiotic resistance, and disease-specific characteristics of this pathogen.

## Materials and methods

### Bacterial strains

A total of 75 strains were included in this retrospective study. Sixty-three strains had been isolated from clinical specimens of patients suffering from acute or persistent focalized Q fever. No patient objected the use of their strain for research purpose. Another 12 strains were isolated from ticks or animals. Among the 75 studied genomes, eight were obtained from Genbank (strains RSA493, RSA331, Dugway, CbuK_Q154, CbuG_Q212, 3262, MSUGoat_Q177, Z3055). All other genomes had been sequenced and assembled in our laboratory. Among those sequenced in our laboratory, the genomes of four strains were previously published (CB175, CB196, CB185, Schperling).

### Identification of *Coxiella burnetii* strains, growth conditions, and DNA extraction

*Coxiella burnetii* strains isolated in our laboratory were identified using PCR targeting the IS1111 and IS30 repeated insertion sequences. All strains were cultured, grown, and produced on Vero cell monolayers (ATCC CRL 1587) maintained in Minimal Essential Medium (MEM) (Invitrogen, Cergy-Pontoise, France) supplemented with 4% fetal bovine serum (Invitrogen) and 1% L-glutamine (Invitrogen), in a 5% CO_2_ atmosphere at 35°C. After cytopathic effect detection, bacteria were purified by successive centrifugations in order to remove cells. DNA extraction was performed using the automated extraction EZ1 DNA Tissue Kit (Qiagen, Hilden, Germany) according to the manufacturer’s instructions.

### Whole genome sequencing

To investigate the clinical features of *C. burnetii*, we sequenced the genomes of human isolates causing various clinical symptoms, in addition to five strains isolated from ticks and six from animals. Among 67 strains sequenced in our laboratory, 15 had been sequenced using SOLiD 4_Life technology while the remaining 52 were sequenced using the Illumina Miseq technology. For the SOLiD 4_Life sequencing, the paired-end library was constructed from 1 μg of purified genomic DNA. The DNA was fragmented on the Covaris device. Libraries were pooled in equimolar ratios and size selected on the E-Gel iBase system at 240–270 bp. Sequencing was carried out to 50 × 35 bp using SOLiD™ V4 chemistry on one full slide of 96 barcoded projects on an Applied Biosystems SOLiD 4 sequencer. The output read length was as expected 85 bp.

The paired-end strategy was used for the 52 strains sequenced using MiSeq Technology (Illumina Inc., San Diego, CA, USA). The genomic DNA was quantified by a Qubit assay with the high sensitivity kit (Life Technologies, Carlsbad, CA, USA) and was barcoded in order to be mixed with 14 other projects with the Nextera XT DNA sample prep kit (Illumina Inc., San Diego, CA, USA).

Purified, normalized and barcoded DNA were pooled for sequencing on the MiSeq sequencer. The pooled single strand library was loaded onto the reagent cartridge and then onto the instrument along with the flow cell. Automated cluster generation and paired-end sequencing with dual index reads were performed in a single 39-h run in 2 × 250-bp.

### Genome assembly and functional annotation

Prior to assembly, raw data quality was checked with the fastQC tool version 0.11.8^1^ (Supplementary Table 1).

Genomes sequenced with Solid were assembled by mapping using the CLC Genomics Workbench version 7.5 (Qiagen). Genomes sequenced with Illumina Miseq were assembled using SPAdes version 3.10 (Bankevich et al., 2012). All *C. burnetii* genomes were annotated using Prokka version 1.12-beta (Seemann, 2014).

### Genotyping of *Coxiella burnetii* strains

The genotypes from all studied strains were determined using the multispacer sequence typing (MST) method, based on 10 intergenic regions as described previously (Glazunova et al., 2005). Moreover, MST PCR was carried out for new or doubtful MST genotypes (Glazunova et al., 2005).

### Comparison of *Coxiella burnetii* genome contents

We performed a COG (Cluster of Orthologous Groups) analysis on the 75 whole genomes as well as on the core genome. We looked for insertion sequences using the ISfinder web server. We also explored the presence of resistance genes using the RAST server. Moreover, digital DNA-DNA hybridization (dDDH) and OrthoAni analysis were performed on the 75 genomes in order to evaluate the range of genomic similarity between strains (Auch et al., 2010; Lee et al., 2016). Plasmids were determined *in silico* with blast and mapping. Virulence factors were determined using the VFDB database (Chen, 2004). The IslandViewer web server was used to predict genomic islands (Bertelli et al., 2017).

### Phylogenomic and pangenomic analysis

Pangenome analyses along with core and non-core region analyses were performed with Proteinortho version 5, with an amino acid identity percentage and coverage threshold of 40 and 60%, respectively (Lechner et al., 2011). Prokka annotations were provided to Proteinortho as an input. Briefly, core regions were defined as genome sequences present in all 75 genomes, while the non-core regions were defined as those differentially present in studied genomes. Unique proteins in sequenced strains were identified from the orthologous list generated by Proteinortho after manual correction of split genes. We constructed a phylogenomic tree using the core region of the 75 *C. burnetii* genomes rather than few genomic loci. Core-genome CDS were inferred from the pangenome, concatenated and aligned using a custom Python script, and then used to construct a phylogenetic tree with MEGA6. SNPs were extracted from the core genome using the SNP-sites software (Page et al., 2016). Visualizations of phylogenetic trees were produced with FigTree.

### Statistical analysis

Statistical analysis was performed using the SPSS software version 22 and XLSTAT 2019 (Addinsoft, Paris, France).

#### Correlation between genomic features of the 75 genomes

The Spearman correlation coefficient was used to correlate between quantitative variables. We studied the relations between the presence of an efflux pump, the genome size and G + C content.

#### Quantitative principal component analysis

In order to explore the geographic and clinical data that corresponded to the 75 genomes, we conducted a principal component analysis with genomic information (genome size, G + C content, MST and plasmid type), geography, host, and clinical presentation.

#### Logistic (exponential) principal component analysis

Thanks to the 75 genomes sequenced here and the data already analyzed in one of our previous studies (Glazunova et al., 2005; Angelakis et al., 2013), we completed the study (205 strains, Supplementary Table 2) of the association of genotypes defined by MST or plasmids and the country of origin of the strain or clinical presentation (acute Q fever or persistent focalized infection). Excluding quantitative variables (genome size, G + C content) and including only strains without missing data into a logistic (exponential) principal component analysis specifically adapted to the qualitative data (Figure 6).

## Results

### Genome sequences of *Coxiella burnetii* strains

In the present study, we reported the new genome sequences from 63 *C. burnetii* strains.

### Genomic features

The average genome size of *C. burnetii* isolates was 2.006 Mb (ranging from 1.910 to 2.158 Mb), and the mean G + C content was 42.62% (ranging from 42.4 to 42.9%), with a number of contigs ranging from 1 to 282 (Table 1).

**TABLE 1:** General features of the 75 studied *Coxiella burnetii* genomes.

Strain	G + C content	Genome size (pb)	No. of contigs	Accession number	Country	Source	Acute/ Persistent	Disease	MST genotype
3262	42.9	2,093,477	1	CP013667	The Netherlands	Human	Persistent	Endocarditis	33
BRASOV	42.7	1,910,413	70	CP103435	Romania	Human	Acute	Pneumonia	16
CB181	42.6	1,972,104	42	JANTNR000000000	French Guiana	Human	Acute	Pneumonia	17
CB1	42.6	2,009,715	78	JAOXFE000000000	France	Human	Persistent	Endocarditis	1
CB10	42.5	2,041,860	86	JAOXFD000000000	France	Human	Persistent	Aneurysm infection	8
CB111	42.6	2,019,099	72	JAOXFC000000000	France	Human	Persistent	Endocarditis	6
CB118	42.6	2,002,150	75	JAOXFB000000000	France	Human	Persistent	Endocarditis	3
CB119	42.6	1,981,226	47	JAOXFA000000000	Senegal	Human	Persistent	Endocarditis	19
CB121	42.6	1,923,427	157	CP103434	Switzerland	Human	Persistent	Endocarditis	12
CB125	42.5	2,040,530	76	JAOXEZ000000000	France	Human	Persistent	Endocarditis	8
CB13	42.6	1,933,670	142	CP103433	France	Human	Persistent	Endocarditis	11
CB132	42.6	1,934,159	130	CP103432	Belgium	Human	Persistent	Endocarditis	11
CB144	42.6	1,956,889	120	JAOXEY000000000	Senegal	Human	Persistent	Endocarditis	12
CB149	42.7	1,924,741	152	CP103431	Senegal	Tick	NA	NA	6
CB152	42.5	2,030,144	51	JAOXEX000000000	Senegal	Tick	NA	NA	16
CB155	42.6	1,924,716	155	CP103430	United Kingdom	Human	Persistent	Endocarditis	55
CB156	42.6	2,020,453	71	JAOXEW000000000	France	Human	Persistent	Endocarditis	4
CB157	42.8	2,022,809	113	JAOXEV000000000	France	Human	Persistent	Endocarditis	33
CB162	42.5	2,017,686	72	JAOXEU000000000	USA	Human	Persistent	Aneurysm infection	8
CB170	42.6	1,929,924	151	CP103429	USA	Human	Persistent	Bone infection	8
CB171	42.6	2,050,297	94	JAOXET000000000	France	Human	Persistent	Aneurysm infection	4
CB175	42.6	1,989,609	1	HG825990	French Guiana	Human	Others	Endocarditis	17
CB176	42.6	1,970,312	37	JAOXES000000000	France	Human	Persistent	Aneurysm infection	17
CB180	42.6	1,944,582	112	CP103428	France	Human	Persistent	Bone infection	18
CB184	42.8	2,026,895	120	JAOXER000000000	France	Human	Persistent	Endocarditis	32
CB185	42.6	1,989,218	282	GCA_000470495	France	Human	Persistent	Miscarriage	33
CB190	42.5	2,018,107	68	JAOXEQ000000000	France	Human	Persistent	Aneurysm infection	8
CB192	42.5	2,020,838	69	JAOXEP000000000	France	Human	Persistent	Endocarditis	8
CB193	42.6	2,025,795	69	JAOXEO000000000	French Guiana	Human	Persistent	Endocarditis	8
CB196	42.6	2,009,497	72	GCA_000820465	Saudi Arabia	Human	Persistent	Endocarditis	4
CB198	42.6	2,003,740	77	JAOXEN000000000	France	Human	Acute	Pericarditis	1
CB2	42.8	2,031,973	117	JAOXEM000000000	France	Human	Persistent	Endocarditis	13
CB201	42.6	2,021,166	70	JAOXEL000000000	France	Human	Persistent	Aneurysm infection	4
CB202	42.5	2,009,975	86	JAOXEK000000000	France	Human	Persistent	Endocarditis	6
CB204	42.6	2,001,464	76	JAOXEJ000000000	France	Human	Acute	Isolated fever	1
CB207	42.4	2,118,168	274	JAOXEI000000000	France	Human	Acute	Hepatitis	16
CB211	42.5	2,046,524	55	JAOXEH000000000	France	Human	Persistent	Endocarditis	16
CB212A	42.7	1,995,348	71	JAOXEG000000000	France	Human	Persistent	Endocarditis	16
CB212	42.6	1,967,025	40	JAOXEF000000000	France	Human	Persistent	Aneurysm infection	20
CB213	42.8	2,040,940	115	JAOXEE000000000	France	Human	Persistent	Endocarditis	32
CB215	42.9	2,058,005	114	JAOXED000000000	France	Human	Persistent	Endocarditis	13
CB216	42.8	2,031,442	114	JAOXEC000000000	France	Human	Persistent	Endocarditis	33
CB217	42.9	2,060,311	118	JAOXEB000000000	France	Human	Persistent	Endocarditis	33
CB218	42.6	1,968,958	38	JAOXEA000000000	France	Human	Persistent	Endocarditis	20
CB220	42.6	1,969,527	40	JAOXDZ000000000	France	Human	Persistent	Bone infection	20
CB221	42.6	1,969,946	32	JAOXDY000000000	France	Tick	NA	NA	16
CB222	42.5	2,018,652	91	JAOXDX000000000	France	Human	Persistent	Endocarditis	6
CB3	42.6	2,002,831	77	JAOXDW000000000	France	Human	Persistent	Endocarditis	1
CB4	42.5	2,016,251	60	JAOXDV000000000	France	Human	Persistent	Endocarditis	21
CB5	42.8	2,023,365	115	JAOXDU000000000	France	Human	Persistent	Endocarditis	12
CB6	42.9	2,050,803	129	JAOXDT000000000	France	Human	Persistent	Endocarditis	12
CB65	42.6	2,038,254	80	JAOXDS000000000	France	Human	Persistent	Endocarditis	9
CB68	42.6	1,928,614	149	CP103427	France	Mouse	NA	NA	9
CB69	42.9	2,051,941	115	JAOXDR000000000	France	Human	Persistent	Endocarditis	13
CB74	42.7	2 004 352	114	JAOXDQ000000000	France	Human	Persistent	Endocarditis	33
CB80	42.6	1,928,715	192	CP103426	France	Human	Persistent	Endocarditis	33
CB85	42.6	1,957,952	118	JAOXDP000000000	France	Human	Persistent	Endocarditis	33
CB9	42.5	2 024 753	74	JAOXDO000000000	France	Human	Persistent	Endocarditis	8
CB94	42.4	2,068,894	176	JAOXDN000000000	France	Human	Acute	Hepatitis	16
CbuG-Q212	42.6	2,008,870	1	CP001019	Canada	Human	Persistent	Endocarditis	21
CbuK-Q154	42.7	2,063,100	1	CP001020	USA	Human	Persistent	Endocarditis	8
DogUtad	42.5	1,956,891	76	CP103425	Canada	Animal	Dog	NA	21
Dugway	42.4	2,158,758	1	CP000733	USA	Animal	Rodent	NA	60
IXO	42.6	1,974,499	67	JAOXDM000000000	Slovakia	Tick	NA	NA	23
CB225	42.6	2,002,796	78	JAOXDL000000000	France	Human	Acute	Hepatitis	1
CB220B	42.6	1,972,402	47	JAOXDK000000000	France	Human	Persistent	Bone infection	20
MSU_Q177	42.7	2,063,021	1	CP018150	USA	Goat	NA	NA	8
NM-I	42.7	1,995,281	1	NC_002971	USA	Tick	NA	NA	16
CB224	42.6	2,032,061	72	JAOXDJ000000000	France	Human	Persistent	Bone infection	8
PokerCat	42.5	2,030,687	68	JAOXDI000000000	Canada	Cat	NA	NA	21
Q229	42.5	1,975,920	41	JAOXDH000000000	Canada	Human	Persistent	Endocarditis	21
Henzerling RSA331	42.8	2,016,427	1	CP014559	Italy	Human	Acute	Pneumonia	18
Schperling	42.6	2,004,282	73	GCA_002634065	Kyrgyzstan	Human	Chronic	Fever	2
Z2534	42.9	2,056,819	115	JAOXDG000000000	Austria	Goat	NA	NA	32
Z3055	42.6	1,995,457	1	LK937696	Germany	Ewe	NA	NA	33

### Genotyping

The 75 studied isolates belonged to 22 different MST genotypes. One new MST genotype was identified in this study (MST 60), and the first genome of a pre-identified MST was sequenced (MST55). The most frequent MST genotypes were MST 8 and 33 (11 and 8 strains, respectively). In contrast, the less represented genotypes were MST 19, 3, 23, 55, and 60, with only one strain each.

### Plasmid analysis and resistance genes of *Coxiella burnetii* isolates

**FIGURE 1:**
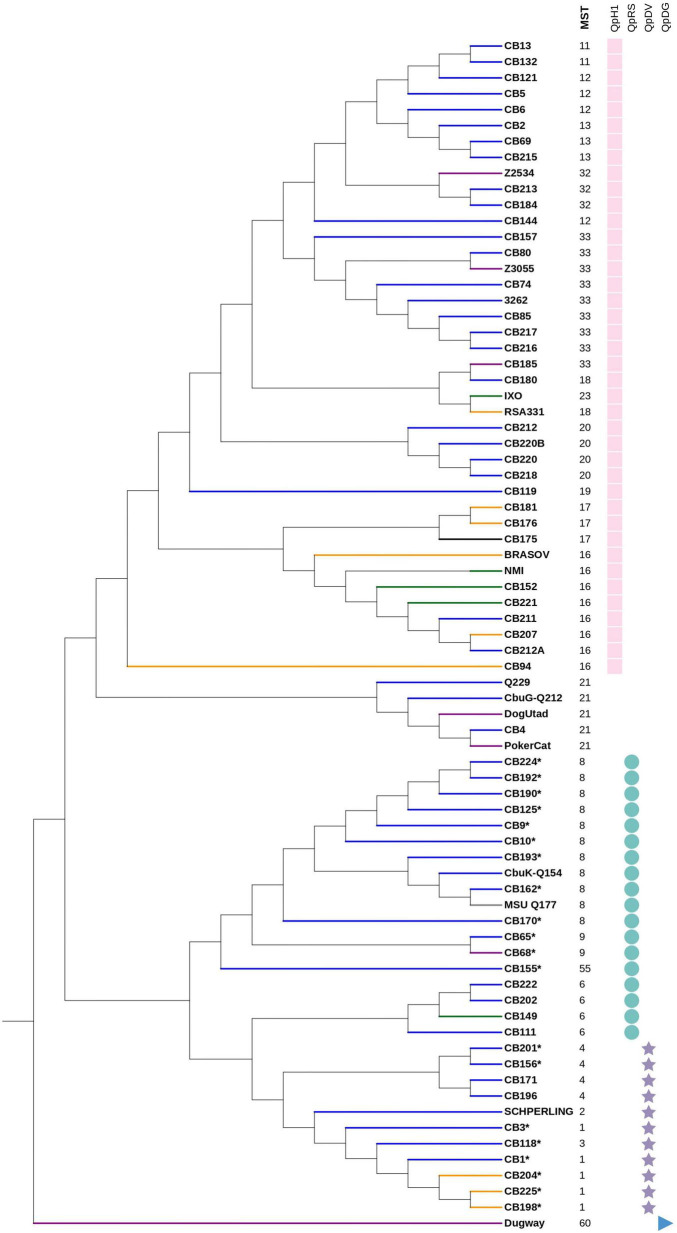
Phylogenetic tree of 75 *Coxiella burnetii* strains using core genome regions, using the Maximum Likelihood method within MEGA6. Branch color indicates the associated disease. Blue branch: persistent focalized Q fever, yellow branch: acute Q fever, green branch: tick isolate, purple branch: animal isolate, black branch: others, gray branch: no data available. *: presence of an efflux pump. On the **right panel:** the first column represents the MST genotype, the remaining columns represent the presence of plasmids with each symbol indicating a plasmid type.

Four types of plasmids were found in 70 out of the 75 studied genomes, while five strains were plasmidless (Figure 1). Among these 70 strains, 40, 18, and 11 strains exhibited the QpH1, QpRS, and QpDv plasmids, respectively. In addition, the Dugway strain was the only strain to exhibit the QpDg plasmid.

Beta-lactam and fluoroquinolone resistance-coding genes were found in all strains. Nineteen strains exhibited a multidrug efflux pump coupled to Na^+^ and belonging to the MATE family. Regarding virulence factors, genes coding for the secretion system type IVB, were found in the 75 studied genomes.

### Phylogenomic analysis

Based on the clustering analysis, the strains could be divided into 13 clusters (Figure 1). The number of strains detected in each phylogenetic cluster along with country of detection, MST genotype, exhibited plasmid and type of infection is summarized in Table 2. This phylogenetic tree is consistent with that obtained from MST sequence analysis (Supplementary Figure 1). Strains exhibiting the same plasmid type were grouped together. In addition, plasmidless strains were clustered into one isolated group (MST21).

**TABLE 2:** Summary of phylogenetic analysis of 75 *Coxiella* genomes.

Phylogenetic cluster	No. of strains	Countries	MST genotypes	Plasmid	Infection types	Clinical manifestation
1	20	France, Belgium, Switzerland, Austria, Germany, and Senegal	11, 12, 13, 32, 33	QpH1	Persistent	Primarily endocarditis
2	4	France, Italy, and Slovakia	18, 23	QpH1	Persistent	Pneumonia and bone infection
3	4	France	20	QpH1	Persistent	Bone infection, aneurysm, endocarditis
4	1	Senegal	19	QpH1	Persistent	Endocarditis
5	10	France, French Guiana, Romania, USA, and Senegal	16, 17	QpH1	Acute and Persistent	Endocarditis, hepatitis, pneumonia, aneurysm
6	1	France	16	QpH1	Acute	Hepatitis
7	5	Canada France	21	None	Persistent	Endocarditis
8	11	France, French Guiana, and the USA	8	QpRs	Persistent	Bone infection, aneurysm, endocarditis
9	2	France	9	QpRs	Persistent	Endocarditis
10	1	UK	55	QpRs	Persistent	Endocarditis
11	4	France, Senegal	6	QpRs	Persistent	Endocarditis
12	11	France, Saudi Arabia, and Italy	1, 2, 3, 4	QpDv	Acute and persistent	Endocarditis, hepatitis, isolated fever, pericarditis, fever, aneurysm infection
13	1	USA (Rodent)	60	QpDg	NA	NA

### Comparative genome analysis and genomic characteristics

Comparative genome analyses were performed for all genomes. In order to determine the range of intra-species genomic similarity, we used dDDH and OrthoANI among strain pairs. dDDH values ranged from 74.5% between strains CB85 and CB3262, to 100% (Supplementary Table 3). OrthoANI values ranged from 99.37% between strain Z3055 and CB65, to 100% (Supplementary Table 4). The 100% values were always observed between strains from the same MST genotype and plasmid type.

Regarding the insertion sequences, the 75 genomes contained the IS1111C, IS1111A, and ISCbu1 insertion sequences. As most genomes were draft sequences, we were not able to determine the number of IS sequences.

**FIGURE 2:**
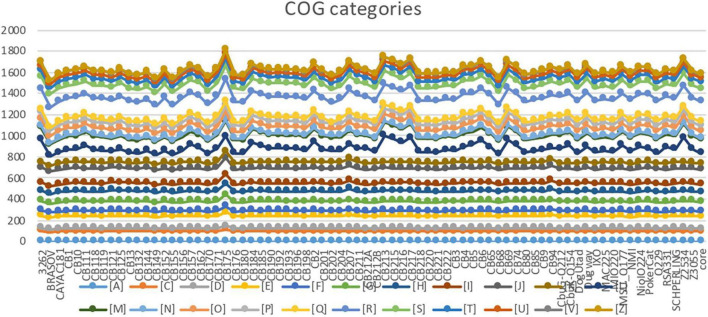
Distribution of genes into cluster of orthologous groups (COG) categories. J: translation, A: RNA processing and modification, K: transcription, L: Replication, recombination and repair, B: Chromatin structure and dynamics, D: Cell cycle control, mitosis and meiosis, Y: Nuclear structure, V: Defense mechanisms, T: Signal transduction mechanisms, M: Cell wall/membrane biogenesis, N: Cell motility, Z: Cytoskeleton, W: Extracellular structures, U: Intracellular trafficking and secretion, O: Post-translational modification, protein turnover, chaperones, C: Energy production and conversion, G: Carbohydrate transport and metabolism, E: Amino acid transport and metabolism, F: Nucleotide transport and metabolism, H: Coenzyme transport and metabolism, I: Lipid transport and metabolism, P: Inorganic ion transport and metabolism, Q: Secondary metabolites biosynthesis, transport, and catabolism, R: General function prediction only, S: Function unknown.

The COG analysis showed a homogenous distribution of gene categories among most genomes (Figure 2). Seventeen genomes had significantly higher numbers of genes in three COG categories. Strain CB175 (strain Guyane) showed the highest number of genes classified in the C to Z COGs categories (Figure 2). Strains CB207 and CB94 had a similar COG profiles with higher numbers of genes in the G to Z COG categories. Similarly, strains CB213, Z2534, CB217, CB215, CB69, CB6, 3262, CB2, CB216, CB184, Dugway, CB5, CB157, and CB74 had higher numbers of genes in L to Z categories (Figure 2).

### *Coxiella burnetii* pangenome

The pangenomic analysis identified a total of 4,501 genes, including 1,211 core genes, and 3,290 accessory genes, including 1,344 unique genes that were only present in one strain. The number of unique genes per strain ranged from 7 to 212. The CB175 and Dugway strains had the highest numbers of unique genes (212 and 140, respectively). Among unique genes, five were likely to be acquired from members of the phylum *Chlamydiae*. Strain CB5 presented three unique genes found in *Chlamydia abortus*, a species that causes fetal death in mammals. Of these genes, one coded a transketolase, and the other two are hypothetical proteins. Strain CB193 exhibited a unique gene coding an hypothetical protein also present in *Chlamydia trachomatis*. Strain Dugway presented one gene found in *Parachlamydia acanthamoebae* and coding an aminoglycoside phosphotransferase, an enzyme known to cause resistance to this antibiotic family.

**FIGURE 3:**
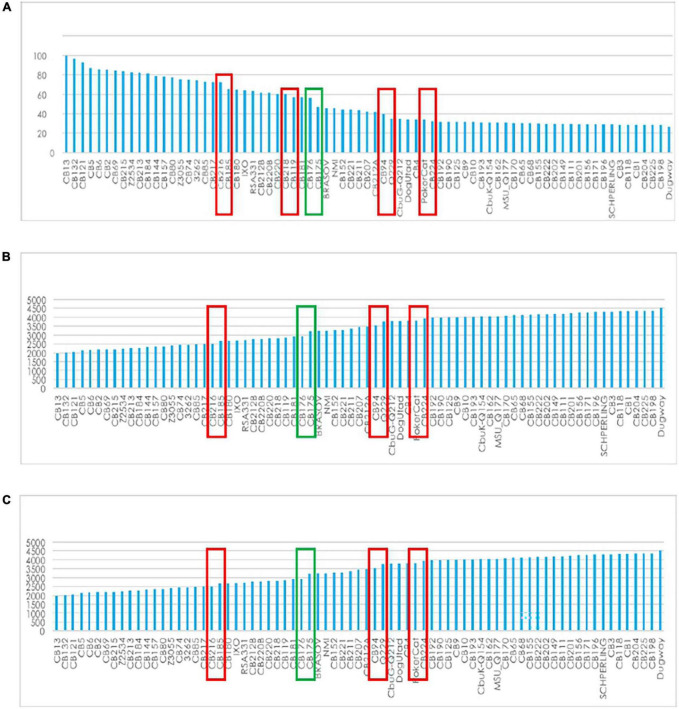
Gene variation. **(A)** Core/pangenome ratio **(B)**: total genes; **(C)** core gene variation with the addition of new genome sequences.

**FIGURE 4:**
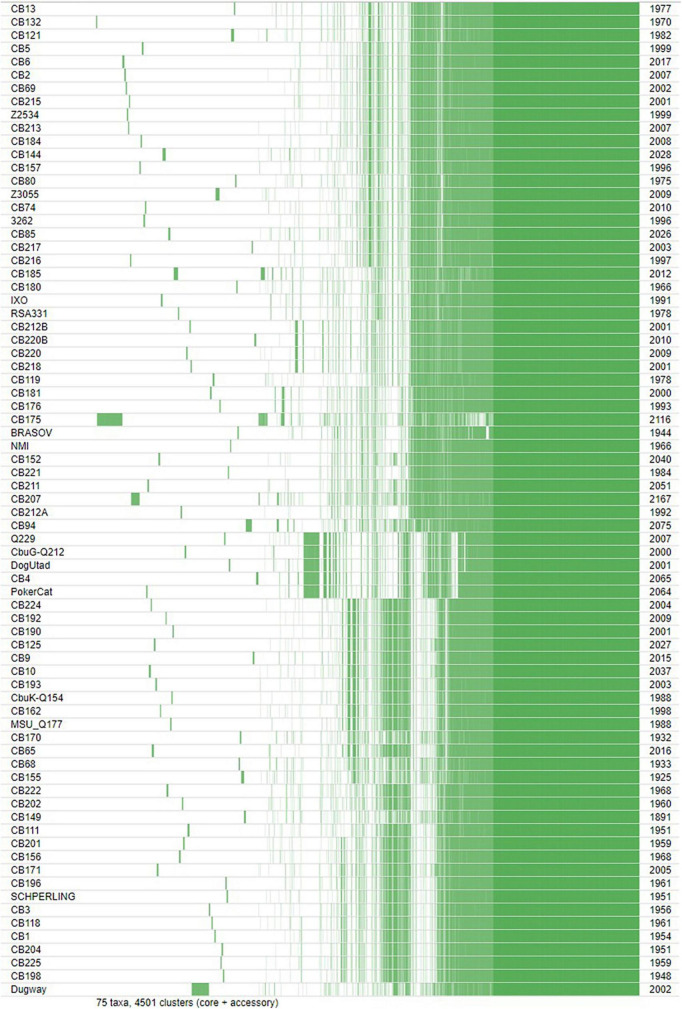
Presence/absence gene matrix of the 75 studied strains.

The core genome length was 1,789,492 bp with a G + C content of 42.7% and 10,192 SNP’s. The variations of core genes, core genome/pangenome ratio and of the total numbers of genes with the addition of new genome sequences is represented in Figure 3. Within each of the above-defined phylogenetic clusters (Figure 1), the contribution of the core genome to the complete gene repertoire of sequenced *C. burnetii* strains ranged from 72 to 95%. However, when this analysis was performed on the 75 genomes, this core genome/pangenome percentage dropped to 27%. Figure 3 shows that almost all the important core/pangenome percentage shifts occurred between MST groups, in addition to an important variation for CB175. The presence/absence gene matrix (Figure 4) showed similar profiles in strains belonging to a given MST genotype. Genome plasticity was clearly between different MST genotypes. Genomic islands which are defined as clusters of genes of probable horizontal origin were found in all strains and in the virulent CB175 Guyanese strain.

### Statistical analysis

#### Correlation between genomic features of the 75 genomes

A significant correlation was found between the presence of the MATE family efflux pump and the G + C content (*p*-value = 0.001). A lower G + C content was correlated with the presence of an efflux pump.

The relationship between genome size and G + C content was examined in these strains, and no significant correlation was found.

#### Quantitative principal component analysis

**FIGURE 5:**
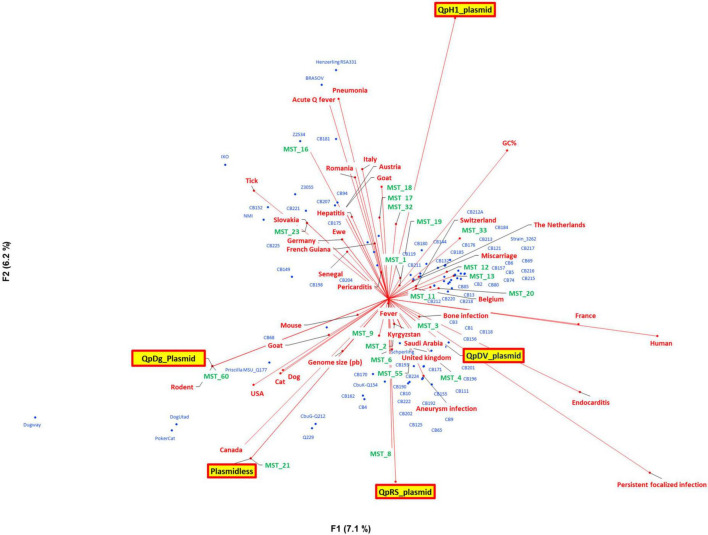
Principal component analysis with genomic information [genome size, G + C content, multispacer sequence typing (MST), and plasmid type], geography, host, and clinical presentation.

This preliminary exploratory analysis suggested non-random associations between genotypes and plasmid types, animal or human host, geography, and clinical symptoms (Figure 5). For example, MST 60, which corresponds to the plasmid QpDG, has only been isolated once from a rodent but never from humans. Plasmidless strains were associated with MST21 and Canada.

**FIGURE 6:**
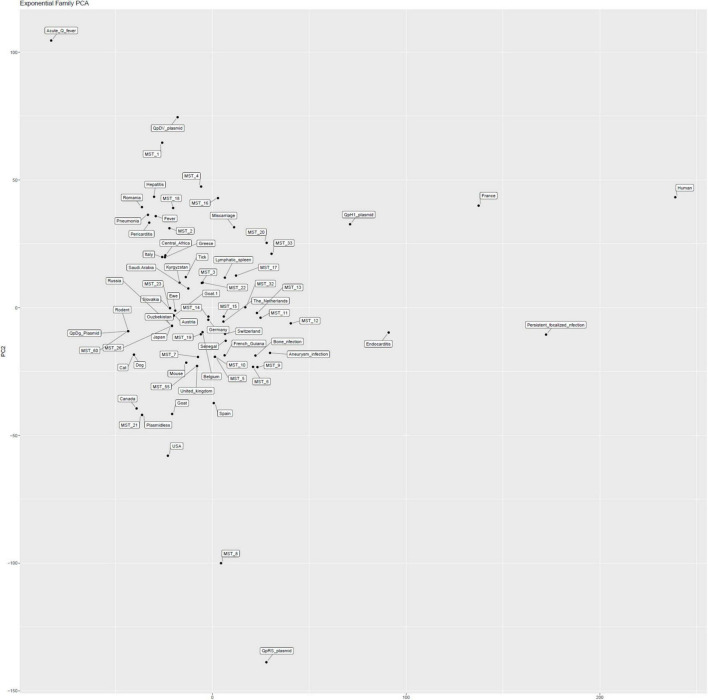
Logistic (exponential) principal component analysis specifically adapted to the qualitative data.

#### Logistic (exponential) principal component analysis

**FIGURE 7:**
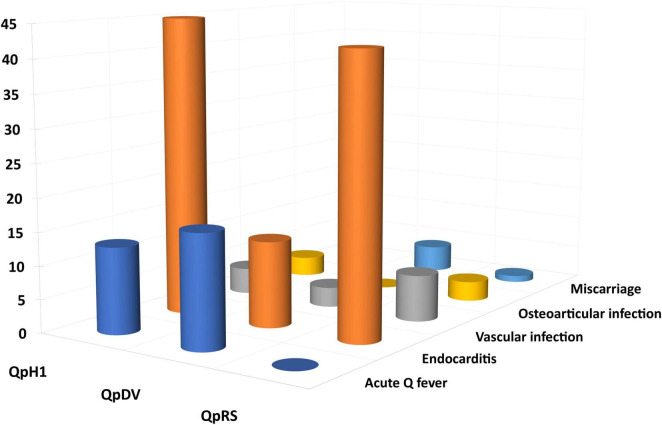
Distribution between plasmids and clinical presentation.

**FIGURE 8:**
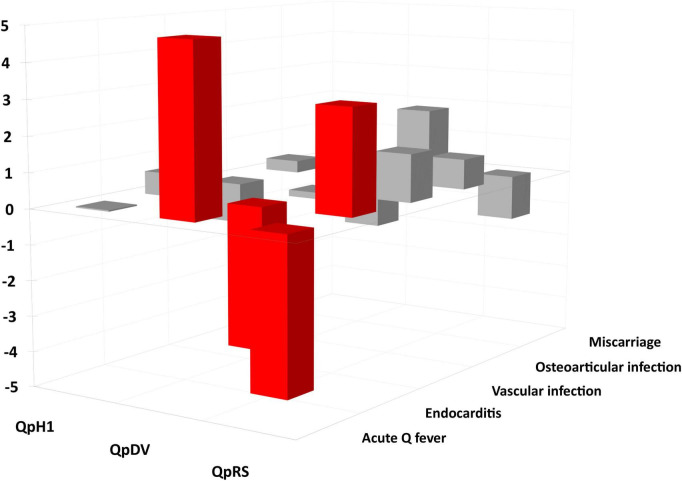
Plasmids and clinics residual analysis. Red: significant difference (*p*-value < 0.05).

We observed that the dataset mostly consisted of endocarditis cases from France (most strains were isolated from cardiac valves from French patients). The population of strains was organized into four groups identified by plasmids. QpDG was associated with animals such as rodents, cats, dogs, and MST60 (Figure 6). QpDV and MST1 were associated with acute Q fever (Figure 6). The association between QpDV and acute Q fever was also definitively confirmed in Tables 3, 4 and Figures 6–8. The QpH1 plasmid was rather associated with endocarditis in France. QpRS had an opposite behavior and was associated with the absence of acute Q fever, and this was consistent among all analyses (Tables 3, 4 and Figures 5–8). Pericarditis, pneumonia, hepatitis, and fever were associated with acute Q fever (Figures 5, 6). Cat, dog, goat, mouse, and rodent strains grouped together (Figures 5, 6). Strains from Canada and the USA were mainly animal strains. These two PCAs (Pearson and logistic) prompted us to formally confirm an association between genotype and geography and clinical presentation. Statistical association with the host was not further tested because most strains [178/205 (86.8%)] were isolated from humans.

**TABLE 3:** Significance by cell.

Plamsid	Acute Q fever	Endocarditis	Vascular infection	Osteoarticular infection	Miscarriage
QpH1	>	>	<	>	<
QpDV	>	<	<	<	>
QpRS	<	>	>	>	<

Fisher’s exact test. Values displayed in gray cells were significant at the level alpha = 0.05. XLSTAT2019 (Addinsoft, Paris, France).

**TABLE 4:** Residuals (adjusted).

	Acute Q fever	Endocarditis	Vascular infection	Osteoarticular infection	Miscarriage
QpH1	0.036	0.65	–1.14	0.35	–0.32
QpDV	**4.77**	−**4.06**	–0.18	–1.38	1.82
QpRS	−**4.32**	**2.95**	1.36	0.87	–1.29

Values displayed in bold are significant at the level alpha = 0.05. XLSTAT2019 (Addinsoft, Paris, France).

#### Genotypes and geography

Among the strains with country of origin and MST information, we were able to identify many significant associations between genotypes and countries of origin (Supplementary Table 2). The strongest associations observed were between MST21 (without plasmid) and Canada (*n* = 5, Pearson coefficient 0.84, *p* < 0.05), between MST33 and The Netherlands (*n* = 10, 0.67, *p* < 0.05), and between MST17 and French Guyana (*n* = 3, 0.57, *p* < 0.05), thus confirming the existence of *C. burnetii* geotypes (Table 5).

**TABLE 5:** Associations of multispacer sequence typing (MST) and countries.

Country	Number of strains from this country	MST	Pearson’s correlation coefficient
Canada	5	21	0.84
The Netherlands	10	33	0.67
French Guiana	3	17	0.57
Russia	5	7	0.57
Russia	5	23	0.57
Senegal	4	19	0.50
Romania	3	18	0.42
USA	10	60	0.37
France	147	1	0.24
Senegal	4	6	0.20
USA	10	8	0.19
France	147	20	0.17
USA	10	16	0.17
Spain	3	4	0.16
Spain	3	8	0.16

Only significant association according to Pearson correlation test are shown (*p* < 0.05). Country with only one or two strains were excluded.

#### Genotypes and acute versus persistent focalized infections

Among 173 human strains for which the plasmid type and clinical presentation was available, we found a significant difference between strains. Strains with a QpRS plasmid [0/55 (0%)] and strains without plasmid [0/3 (0%)] were never isolated from patients with acute Q fever. QpDV and QpH1 plasmid-positive strains were isolated from both patients with acute Q fever and persistent focalized infections. Acute Q fever corresponded to 17/44 (38.6%) QpDV strains but only 13/71 (18.3%) QpH1 strains. The difference in proportion was very significant between the four types (Chi-square = 26.1; *p* < 0.0001). When analyzing case significance with the Fisher’s exact test, non-plasmid strains and QpRS strains were significantly associated with persistent focalized infections (*p* < 0.05); QpRS strains were significantly associated with the absence of acute Q fever; QpDV strains were significantly associated with more acute Q fever and fewer persistent focalized infections (*p* < 0.05–Figures 7, 8).

After selecting the human strains for which the plasmid and clinical presentation were available, we excluded plasmids represented by less than four strains (plasmidless strains, *n* = 3) and clinical forms represented by less than four strains (pneumonia, lymphatic infection). We therefore included 158 strains in this analysis. The distribution between plasmids (QpH1, QpDV, and QpRS) and clinical presentations (acute Q fever, endocarditis, vascular infection, miscarriage, osteoarticular infection) was described in Figure 7 and Table 6.

**TABLE 6:** Association between plasmid type and clinical presentation.

Plasmid	Acute Q fever	Endocarditis	Vascular infection	Osteoarticular infection	Miscarriage
QpH1	13	45	4	3	3
QpDV	17	13	3	0	4
QpRS	0	42	7	3	1

Using the chi-square test, the Fisher’s exact test per case (Table 3) and residual analysis [Table 4 and Figure 8; XLSTAT 2019 (Addinsoft, Paris, France)], we found that clinical presentation and plasmid type were not independent (Observed Chi-square 38.4, critical value 15.5, *p* < 0.0001). Strains with a QpDV plasmid were associated with more acute Q fever, less endocarditis and less osteoarticular infection. In addition, QpRS strains were not associated with any acute Q fever (0/53) but were associated with endocarditis. There was neither significant association for the other clinical forms nor for QpH1 with any clinical presentation. The QpRS plasmid was never identified in any acute Q fever strain but no specific geographic association was identified as the 63 QpRS plasmid-positive strains in our study were identified in several countries (49 in France, six in the USA, two in Spain, two in Russia, one in French Guiana, one in Senegal, one in the United Kingdom, one in Uzbekistan).

## Discussion

We performed a pangenomic analysis of 75 *C. burnetii* isolates to understand better the evolutionary traits of their genomes. Focus was given on the links between genome content, epidemiological characteristics and pathogenicity. To our knowledge, the present work is the largest study on the association of *C. burnetii* strains and clinical presentation. Eighty-five percent of studied strains were isolated from human samples, including 69% from patients suffering from persistent focalized Q fever, and 59% from France. We first performed a phylogenomic analysis. Regardless of whether the studied genomes were complete or draft genomes, or whether they were sequenced using Illumina Miseq or Solid, clustering of the strains was consistent with the MST genotyping and the core genome SNP analysis, thus confirming the genotype-specific genetic differences found in other studies (Supplementary Figure 2; Kuley et al., 2017). The reliability of genotyping was also demonstrated during the Dutch outbreak (Kuley et al., 2017). Thus, the use of MST genotyping as a sensitive and less-time consuming tool should be considered in the future as a discriminatory tool for outbreak investigation and molecular epidemiology. In this study we identified a new MST genotype (MST60-Dugway strain) observed in a strain causing an independent cluster. In addition, we observed a concordance between MST genotyping, core genome analysis and plasmid type, which suggests an ancient acquisition of plasmids.

Comparative genomic analysis showed a high degree of genetic similarity between *C. burnetii* strains belonging to the same cluster. The highest values of OrthoANI and dDDH analyses were observed among strains from the same MST and harboring the same plasmid type. The classification of proteins by COG categories showed a homogenous distribution among many strains, especially for categories A, C, D, E, F, G, H, I, J, and K. Similarly, COG profiles of strains from a first phylogenetic cluster represented by four MST genotypes (MST12, MST13, MST32, MST33) exhibited greater gene numbers in the L-Z COG categories. These strains were isolated from Europe and had high G + C contents, numbers of genes and genome sizes than the remaining *Coxiella* genomes, thereby confirming close regional evolutionary relationships of genomic groups as shown in previous studies, notably between UK strains and those from neighboring European countries (Hemsley et al., 2019).

Strain CB175, restricted to French Guiana, exhibited a COG profile as unique as its geographic distribution and virulence (Mahamat et al., 2013). Strain CB175 had the highest number of genes, irrespective of its smaller genome size compared to 67% of strains in this study. Thus, it was suspected that the virulence of this strain be linked to its genome reduction (D’Amato et al., 2015). The two strains CB94 and CB207, isolated from patients with hepatitis, exhibited similar COG profile (G-Z categories; Figure 2), the same MST16 genotype and the same G + C content despite a significant difference in genome size (Table 1). These observations indicate that the pathogenicity and clinical expression of *C. burnetti* strains may be related to their genomic content. The pangenome analysis also demonstrated a high genetic homogeneity among strains from the same MST type, which was in concordance with previous studies (D’Amato et al., 2014; Kuley et al., 2017). It also unveiled a low core/pangenome ratio, strain CB175 being an important contributor to this low value. This strain was demonstrated to have a 6,105-bp genomic deletion and a greater number of gene mutations when compared to strain NM-I (D’Amato et al., 2015). In addition, important core/pangenome percentage shifts were observed between MST groups and in some specific strains. By testing associations between strains characterized in the present study and including data from previous studies (Glazunova et al., 2005; Angelakis et al., 2013), we definitively demonstrated associations between genomic types (MST and plasmid types) and geographic distribution or clinical forms. Therefore MST genotyping may enable screening the strain type during an outbreak, which may play a key role in orientating the patient management and treatment approach. Although some genotypes appeared to be widely distributed, some were clearly specific, notably in French Guiana or during the Dutch outbreak. We also confirmed the association of plasmidless MST21 strains and Canada. This suggests that the geographic distribution may vary greatly among MST genotypes and that specific clonal complexes are associated with explosive outbreaks (MST33 in the Netherlands) or a localized hyperendemicity (MST17 in French Guiana) while other genotypes have broader geographical and temporal distributions, as is the case for MST1. Other studies also demonstrated a preferential associations of some genotypes with specific hosts (Roest et al., 2011) or specific epidemiological situations (Eldin et al., 2017).

Previous studies showed a relation between the presence of QpH1 and QpDV plasmids and acute infections or abortions (Angelakis et al., 2013). In our study, we observed that all isolates causing acute infections harbored either the QpH1 or QpDV plasmids and never the QpRs plasmid, even though these plasmid types are not exclusively found in acute Q fever-associated strains (Eldin et al., 2017). This finding has a clinical impact because our results suggest that QpRS-harboring *C. burnetii* strains cause asymptomatic primary infections. If this is confirmed, it would be impossible to prevent endocarditis or other persistent focused infections after primary infections in patients infected by such strains. This finding was not related to the confounding role of geography as several QpRS strains are found in several countries. Based on these findings, confirmed by genomic and statistical analyses, we assume that strain characteristics play an important role in the clinical manifestations of Q fever.

As previously described, *C. burnetii* possesses an important number of IS sequences in comparison to other intracellular bacteria (Seshadri et al., 2003). We detected IS sequences in all studied genomes which is in concordance with other studies that observed numerous IS sequences in *C. burnetii* genomes, thus conferring a greater genome plasticity to *C. burnetii* in comparison to other intracellular bacteria (Beare et al., 2009; Kuley et al., 2017). Genomic plasticity was clearly demonstrated in Figure 4. The detection of genes present in other intracellular bacteria, as is the case of the unique genes found in strains CB5, CB193, and Dugway, suggests that, during its evolution, *C. burnetii* shared the same host with other intracellular bacteria, thus enabling gene transfers.

Regarding antibiotic susceptibility, rare resistance issues are observed in clinical practice. Genes coding for resistance to betalactams and fluoroquinolones were found in all strains (Spyridaki, 2002; Vranakis et al., 2010). The presence of an efflux pump was observed in some strains that belong to the same branch in the phylogenetic tree. This efflux pump belonging to the MATE family was found in different bacteria such as *Escherichia coli* and *Klebsiella pneumoniae* but has not been associated with any antibiotic resistance to date (Ogawa et al., 2015). Statistical analyses showed a significant correlation between the presence of this efflux pump and a low G + C content, suggesting that it might be acquired *via* horizontal gene transfer, thus reinforcing the idea of genome plasticity and genotype-specific pathogenicity.

In conclusion, genomic analysis enabled us to understand more of the genomic dynamics of *C. burnetii*, its evolution, host adaptation, epidemiology, and clinical profile. In this study we demonstrated that *C. burnetii* exhibits a high genome plasticity and that some of the genomic features are correlated to the clinical outcome, epidemiological characteristics and geographic distribution. Future studies including more animal and human-infecting strains from different MST genotypes and from additional geographical locations will complete our current perspective of *C. burnetii* genomic evolution and pathogenesis.

## Data availability statement

All sequences presented in this study were deposited in GenBank under the accession numbers listed in Table 1.

## Author contributions

RA and P-EF designed the project. RA, HA, JD, AD, AC, ER, KS, and RZ performed the experiments and genome analysis. RA and MM performed the statistical analysis. RA performed the data analysis. RA, MM, and P-EF wrote the manuscript. P-EF, DR, and MM revised the manuscript. All authors contributed to the article and approved the submitted version.

## Footnotes

1
https://www.bioinformatics.babraham.ac.uk/projects/fastqc/
